# Inducible MdAGG lectins in apple immunity toward fire blight: CRISPR/Cas9 validation and their potential for intragenesis approaches

**DOI:** 10.1093/hr/uhaf262

**Published:** 2025-10-03

**Authors:** Antoine Bodelot, Nicolas Dousset, Elisa Ravon, Christelle Heintz, Marie-Noelle Brisset, Alexandre Degrave, Emilie Vergne

**Affiliations:** Université Angers, Institut Agro, INRAE, IRHS, SFR QUASAV, 49000 Angers, France; Université Angers, Institut Agro, INRAE, IRHS, SFR QUASAV, 49000 Angers, France; Université Angers, Institut Agro, INRAE, IRHS, SFR QUASAV, 49000 Angers, France; Université Angers, Institut Agro, INRAE, IRHS, SFR QUASAV, 49000 Angers, France; Université Angers, Institut Agro, INRAE, IRHS, SFR QUASAV, 49000 Angers, France; Université Angers, Institut Agro, INRAE, IRHS, SFR QUASAV, 49000 Angers, France

## Abstract

Fire blight, caused by the bacterium *Erwinia amylovora*, represents a significant threat to apple (*Malus domestica*) production. Currently, only a limited number of genes effectively involved in resistance to *E. amylovora* have been identified. Seeking new resistance candidates, we focused on a multigene family encoding amaranthin-like lectins, which are highly upregulated following chemical elicitation by acibenzolar-*S*-methyl (ASM). These lectins are believed to contribute to downstream defense by promoting bacterial aggregation, which led to their designation as *Malus domestica* agglutinins (MdAGGs). When loss-of-function editions were introduced into *MdAGG* genes, the plant’s ability to mount a fully effective defense response against fire blight upon ASM treatment was compromised, confirming the role of MdAGGs in fire blight resistance. Next, we coupled the *pPPO16* promoter, endogenous to apple and known to be rapidly induced during *E. amylovora* infection, with the coding sequence of MdAGG10 to create apple lines with fire blight-inducible *MdAGG10* expression. Early *MdAGG10* expression in these lines significantly improved resistance to fire blight, and an additional ASM treatment further enhanced this resistance. In summary, we conclude that MdAGGs act as defense genes whose timely expression can provide effective resistance against *E. amylovora*.

## Introduction

Fire blight is a devastating disease caused by the necrogenic bacterium *Erwinia amylovora* (*Ea*), which affects most members of the Rosaceae family, including apple (*Malus domestica*). *Ea* has two major virulence factors. The first one is its type III secretion system (T3SS), which injects effectors into host cells, and these effectors are assumed to be secreted and translocated via this T3SS, i.e. AvrRpt2_Ea_, DspE/A, HopPtoC_Ea_, HrpN, Eop1 and Eop3 [1]. The second factor is exopolysaccharide (EPS), external glycans whose production is correlated to *Ea* virulence [2]. As *Ea* is a Gram-negative bacterium, its cells have an external membrane that also contains lipopolysaccharides (LPS), which are glycolipids protecting the bacterial cells from the oxidative burst launched by the plant during infection [3]. EPS and LPS are known bacterial PAMPs (pathogen-associated molecular patterns), widely conserved components essential for pathogenicity and pathogen survival [4].

As protection practices against fire blight are scarce and often poorly effective [5], the selection of resistant cultivars remains a favored alternative. However, conventional breeding of apple is tedious, especially due to a long juvenile phase [6]. Until now, conventional breeding for fire blight resistance has mostly focused on resistance quantitative trait loci (QTLs), the strongest ones identified in wild *Malus* being on linkage group (LG) 3 of *Malus × robusta 5* [7], on LG10 of *M. fusca* [8] and on LG12 of ‘Evereste’, *M. floribunda 821* [9] and *M. × arnoldiana* [10]. Candidate genes under these QTLs have been assigned as NBS-LRR *FB_MR5* in *Malus × robusta 5* [11]; receptor-like kinase *FB_Mfu10* in *M. fusca* [12], both of which are in the QTL on LG12 of ‘Evereste’ (and *M. floribunda* [13]); and *M. × arnoldiana* [14]. However, bypasses of the resistance gained from the introgression of QTLs from ‘Evereste’ (and *M. floribunda*) and *Malus × robusta* (*FB_MR5* gene) into commercial varieties have already been observed in apple, due to the evolution of the bacterial effectors Eop1 and AvrRPT2_Ea_, respectively [15].

New breeding technologies, including cisgenesis, intragenesis, RNA interference and genome editing mediated by CRISPR-Cas9, are alternatives to conventional breeding [6]. These technologies, however, have rarely been used to improve resistance to fire blight, via R recognition mechanisms or via targeting mechanisms considered to be more durable. Cisgenesis has been used to introduce the *FB_MR5* gene from *Malus × robusta 5* into the susceptible genotype ‘Gala Galaxy’, leading to resistance levels equivalent to those obtained for classically bred descendants [16]. Intragenesis has already been employed to improve scab resistance in apple [17], but not yet for fire blight resistance. Successful knockdown of apple susceptibility genes (S genes [18, 19]) has been performed by RNA interference on the *HIPM* gene [18] or by directed mutagenesis by CRISPR/Cas9 of the *DIPM4* gene [19], both genes encoding proteins that respectively interact with *Ea* effectors HrpN and DspA/E. To the best of our knowledge, no published attempts have been made to improve apple resistance to *Ea* through NBT by targeting defense genes, such as pathogenesis-related (PR) genes, which are involved in downstream mechanisms of pathogen recognition by R proteins or pattern recognition receptors (PRRs) and subsequent signal transduction (pattern-triggered immunity, PTI [20]).

The candidate family we targeted in the present study was identified in previous work on elicitor-induced resistance in apple against *E. amylovora* and proposed as a new class of PR proteins [21, 22]. In particular, the chemical elicitor ASM upregulates thousands of genes in apple, including a multigene family partly organized as tandem array genes encoding lectins that have been termed *Malus domestica* agglutinins (MdAGGs [22]). Lectins are proteins with at least one non-catalytic site able to reversibly bind mono- or oligosaccharides [23]. Plant PRRs harboring extracellular lectin domains that recognize specific carbohydrates of pathogens and trigger subsequent defense responses have received extensive interest [24]. In contrast, defense-inducible lectins not related to pathogen recognition and subsequent signalization are less studied, although they can bind carbohydrate patterns and act as key players in plant defense processes through different pathways [25]. According to the classification of Van Damme *et al.* [26], MdAGG1–18 are defense-inducible merolectins composed of a unique amaranthin-like carbohydrate-binding domain. The apple genome also encodes nine other amaranthin-like lectins, MdDiAGG1–9, composed of two amaranthin-like domains and an additional aerolisin/ETX pore-forming domain for three of them: MdDiAGG5, 6 and 7 [22]. *MdDiAGG*s are constitutively expressed, unlike *MdAGG*s, and *MdAGG10*, which is one of the most ASM-induced genes and aligns best with the *MdAGG* consensus sequence [22], has been studied further. Agglutination of *Ea* cells by MdAGG10 has been demonstrated *in vitro*, involving electrostatic interactions with the negatively charged bacterial polysaccharides EPS and LPS. Moreover, *Ea* actively downregulates the expression of *MdAGG* genes and evades MdAGG10-driven cell aggregation thanks to the secretion of EPS [21]. *MdAGG*s are therefore assumed to be good candidate defense genes for improving apple PTI against *Ea*. However, there is still no functional evidence supporting the involvement of MdAGGs in apple resistance to *Ea*, as the constitutive overexpression of *MdAGG10* in the susceptible ‘Gala’ genotype did not improve resistance to *Ea* [27]. Moreover, constitutive expression of *MdAGG10* is associated with growth and development defaults of apple transgenic lines [27]. Associating *MdAGG* sequences with an *Ea*-inducible promoter appears to be an alternative to the apparent toxicity of the constitutive accumulation of these proteins, and its possible negative impact on resistance. From this perspective, the promoter of the endogenous apple *PPO16* gene, known to be activated by *Ea* infection in a rapid and sustained manner [28], was considered a valuable candidate.

The present study aimed to determine if *MdAGG*s genes are effectively involved in apple resistance against *Ea*. Thanks to CRISPR/Cas9, now widely mastered in apple [6], we evaluated the consequences of *MdAGG* knockdown for ASM-induced resistance to *Ea*. Using the intragenic association between the fire blight-inducible *pPPO16* promoter and the coding sequence of *MdAGG10* allowed us to study the effect of *Ea*-driven expression of *MdAGG10* on disease progression. From a methodological perspective, we evaluated the use of *in vitro* apple shoots for preliminary phenotyping of transformed lines, and a qPCR-based method to calculate the overall editing rate of edited lines.

## Results

### Generation of transgenic lines

A total of 419 leaf explants were inoculated with *Agrobacterium tumefaciens* containing the binary vector with the CRISPR-MdAGGAll construct (T-DNA in Supplementary Data Fig. S1a) targeting supposedly all of the *MdAGG* family members in ‘Gala’ (Table 1). The five kanamycin-resistant regenerants that survived 6 months of micropropagation were analyzed by PCR to confirm transformation and absence of *A. tumefaciens* contamination (Supplementary Data Fig. S2a). Amplification using primers I (Supplementary Data Table S1) designed for the *EF1α* gene was used as a positive PCR control, plasmid DNA extracted from *A. tumefaciens* strain containing the CRISPR-MdAGGAll construct was used as a positive control of T-DNA presence, and genomic DNA extracted from a non-transgenic ‘Gala’ was used as a negative control of T-DNA-presence. K primers (Supplementary Data Table S1) amplifying 23S ribosomal RNA from *A. tumefaciens* showed that all tested lines were free from *A. tumefaciens* contamination. Amplification using J primers (Supplementary Data Table S1), which target the *nptII* selection gene, confirmed that all five lines were true transgenics containing the selectable marker. Finally, amplification using primers B and L (Supplementary Data Table S1), which respectively target gRNA sequences and the Cas9 coding sequence, demonstrated that all lines had integrated the full construct. Considering the initial number of explants (419), the transformation rate was therefore 1.19%. The five lines were called *mdagg-1* to *mdagg-5* and further analyzed.

**Table 1:** Information on the gRNA1- and gRNA2-targeted DNA zones in the GDDH13 genome and the two phases of the ‘Gala’ genome. gRNAs target all *MdAGG* members of the family. gRNA1 and 2 were designed on *MdAGG10* (MD10G1027210) on the GDDH13 genotype.

	Information on GDDH13 genotype^b^					
						
Gene name^a^	Gene ID		Genome localization	Positions of gRNA1 ‘off-targets’ with 0, 1 or 2 mismatches^d^	gRNA1 mismatches	gRNA1 edit
MdAGG1	MD10G1026610	Cluster Chr. 10 250 kb	Chr10:3293389.0.3293886 (− strand)	Position: Chr10:3293855–3293877:+	1	Yes
MdAGG2	MD10G1026620		Chr10:3296592.0.3297089 (− strand)	Position: Chr10:3297058–3297080:+	1	Yes
ABS1	Intergenic region		_	Position: Chr10:3307773–3307795:+	1	Yes
MdAGG3	MD10G1026810		Chr10:3322816.0.3323313 (− strand)	Position: Chr10:3323282–3323304:+	1	Yes
MdAGG4	MD10G1026820		Chr10:3325980.0.3326477 (− strand)	Position: Chr10:3326446–3326468:+	2	Yes
MdAGG5	MD10G1027010		Chr10:3339497.0.3339994 (− strand)	Position: Chr10:3339963–3339985:+	2	Yes
MdAGG6	MD10G1027020		Chr10:3349989.0.3350522 (− strand)	Position: Chr10:3350491–3350513:+	0	Yes
MdAGG7	MD10G1027025		Chr10:3362283.0.3362780 (−strand)	Position: Chr10:3362749–3362771:+	0	Yes
MdAGG8	MD10G1027030		Chr10:3384282.0.3384779 (− strand)	Position: Chr10:3384748–3384770:+	0	Yes
ABS2	Intergenic region		_	Position: Chr10:3385523–3 385 545:+	2	Yes
MdAGG9	MD10G1027110		Chr10:3423166.0.3423663 (− strand)	Position: Chr10:3423632–3423654:+	0	Yes
MdAGG10	MD10G1027210		Chr10:3455323.0.3455820 (− strand)	targeted gene of gRNA1 (position 10 − strand)	0	Yes
MdAGG11	MD10G1027220		Chr10:3483442.0.3483939 (− strand)	Position: Chr10:3483908–3483 30:+	0	Yes
MdAGG12	MD10G1027230		Chr10:3509319.0.3509813 (−strand)	Position: Chr10:3509785–3509807:+	0	Yes
MdAGG13	MD10G1027240		Chr10:3524494.0.3524991 (− strand)	Position: Chr10:3524960–3524982:+	0	Yes
MdAGG14	MD10G1027250		Chr10:3537095.0.3537592 (− strand)	Position: Chr10:3537561–3537583:+	1	Yes
MdAGG15	MD10G1027260		Chr10:3553278.0.3553775 (− strand)	Position: Chr10:3553744–3553766:+	0	Yes
MdAGG16	MD06G1232610		Chr06:36368957.0.36369454 (− strand)	Position: Chr06:36369423–36369445:+	2	Yes
MdAGG17	MD05G1031110		Chr05:5050912.0.5051415 (+ strand)	Position: Chr05:5050921–5050943:−	2	Yes
MdAGG18	MD05G1031120		Chr05:5072620.0.5073123 (+ strand)	Position: Chr05:5072629–5072651:−	2	Yes
MdDiAGG1	MD06G1232620		Chr06:36378219.0.36379187 (− strand)	_	_	_
MdDiAGG2	MD06G1232630		Chr06:36397896.0.36398933 (− strand)	_	_	_
MdDiAGG3	MD06G1233110		Chr06:36438595.0.36439497 (+ strand)	_	_	_
MdDiAGG4	MD14G1239550		Chr14:31867464.0.31868432 (− strand)	Position: Chr14:31867930–31867952:+	2	Yes
MdDiAGG5	MD07G1171100		Chr07:24677707.0.24679209 (+ strand)	_	_	
MdDiAGG6	MD01G1104300		Chr01:21733076.0.21734572 (+ strand)	_	_	
MdDiAGG7	MD02G1240000		Chr02:28852956.0.28854545 (+ strand)	_	_	
MdDiAGG8	MD03G1201500		Chr03:27573652.0.27575032 (+ strand)	_	_	
MdDiAGG9	MD14G1241050		Chr14:31980059.0.31981000 (− strand)	_	_	

(Continued)

**Table 1:** *(Continued)*. Information on the gRNA1- and gRNA2-targeted DNA zones in the GDDH13 genome and the two phases of the ‘Gala’ genome. gRNAs target all *MdAGG* members of the family. gRNA1 and 2 were designed on *MdAGG10* (MD10G1027210) on the GDDH13 genotype.

Information on ‘Gala’^c^				Information on GDDH13 genotype^b^			Information on ‘Gala’^c^			
Phase A		Phase B					Phase A		Phase B	
gRNA1 mismatches	gRNA1 edit	gRNA1 mismatches	gRNA1 edit	Positions of gRNA2 ‘off-targets’ with 0, 1 or 2 mismatches^d^	gRNA2 mismatches	gRNA2 edit	gRNA1 mismatches	gRNA1 edit	gRNA1 mismatches	gRNA1 edit
1	Yes	1	Yes	Position: Chr10:3293631–3293653:−	0	Yes	0	Yes	0	Yes
1	Yes	1	Yes	Position: Chr10:3296834–3296856:−	0	Yes	0	Yes	0	Yes
1	Yes	0	Yes	_	_	_	_	_	_	_
1	Yes	1	Yes	Position: Chr10:3323058–3323080:−	0	Yes	0	Yes	0	Yes
2	Yes	2	Yes	Position: Chr10:3326222–3326244:−	0	Yes	0	Yes	0	Yes
2	Yes	2	Yes	Position: Chr10:3339739–3339761:−	0	Yes	0	Yes	0	Yes
0	Yes	0	Yes	Position: Chr10:3350267–3350289:−	1	Yes	1	Yes	1	Yes
0	Yes	1	Yes	Position: Chr10:3362525–3362547:−	1	Yes	0	Yes	1	Yes
0	Yes	_	_	Position: Chr10:3384524–3384546:−	1	Yes	1	Yes	1	Yes
_	_	_	_	_	_	_	_	_	_	_
0	Yes	0	Yes	Position: Chr10:3423408–3423430:−	0	Yes	0	Yes	0	Yes
0	Yes	0	Yes	targeted gene of gRNA2 (position 254 + strand)	0	Yes	0	Yes	0	Yes
0	Yes	0	Yes	Position: Chr10:3483684–3 83 06:−	0	Yes	0	Yes	0	Yes
0	Yes	0	Yes	Position: Chr10:3509561–3509583:−	0	Yes	0	Yes	0	Yes
0	Yes	0	Yes	Position: Chr10:3524736–3524758:−	1	Yes	1	Yes	1	Yes
1	Yes	1	Yes	Position: Chr10:3537337–3537359:−	1	No	1	No	1	No
0	Yes	0	Yes	Position: Chr10:3553520–3553542:−	0	Yes	0	Yes	0	Yes
_	_	2	Yes	Position: Chr06:36369199–36369221:−	1	Yes	_	_	1	Yes
2	Yes	2	Yes	Position: Chr05:5051145–5051167:+	1	No	1	No	1	No
2	Yes	2	Yes	Position: Chr05:5072853–5072875:+	1	No	1	No	1	No
_	_	_	_	_	_	_	_	_	_	_
_	_	_	_	_	_	_	_	_	_	_
_	_	_	_	_	_	_	_	_	_	_
_	_	2	Yes	_	_	_	_	_	_	_
_	_	_	_	_	_	_	_	_	_	_
_	_	_	_	_	_	_	_	_	_	_
_	_	_	_	_	_	_	_	_	_	_
_	_	_	_	_	_	_	_	_	_	_
_	_	_	_	_	_	_	_	_	_	_

On the two genotypes, gRNA1 targets all *MdAGGs* with more or less mismatches, but also the off-target coding sequence *MdDiAGG4* (MD14G1239550) with two mismatches. On the two genotypes, gRNA2 targets all other *MdAGG*s with more or less mismatches except MdAGG14 (MD10G1027250), MdAGG17 (MD05G1031110) and MdAGG18 (MD05G1031120); it has no predicted off-target*.* Mismatch tolerance is considered effective (‘Yes’) for up to two base mismatches [29], occurring between positions 20 and 4 relative to the PAM. Underline characters (_) indicate non-off-target sequences.

a ABS1 and 2 are intergenic regions not encoding MdAGGs or MdDiAGGs [22].

b From https://iris.angers.inra.fr/gddh13.

c BLAST on sequences from genomes ‘Malus_domestica_Gala.altA’ (phase A) and Malus_domestica_Gala.altB” (phase B) [30].

d Positions from http://crispor.gi.ucsc.edu/.

**Figure 1:**
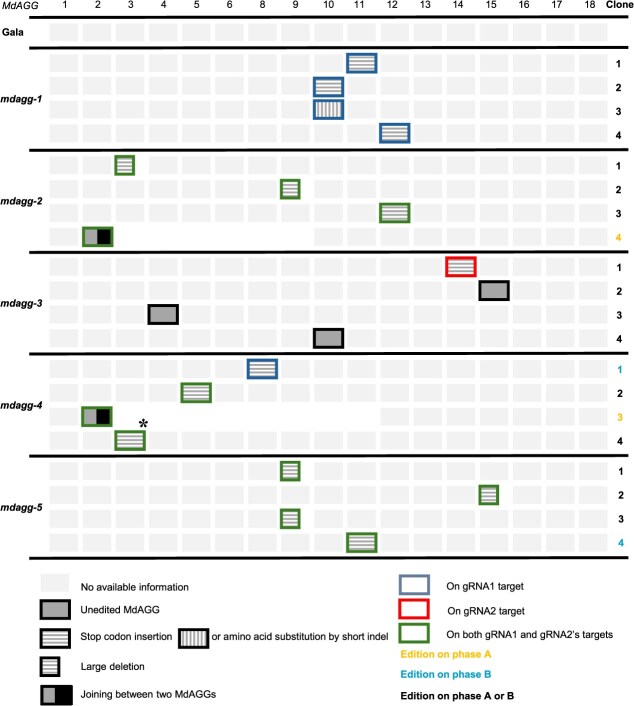
Dual guide-RNA gene edition strategy leads to *MdAGG* gene knockdown in apple. Four cloned sequences from each of the five independent *mdagg*-edited apple mutants were analyzed. Each box represents one *MdAGG* sequence. Unedited *MdAGG* sequences are represented by a filled box, short indels leading to frameshift mutations protein sequence (respectively early insertion of stop codon) are represented by boxes with vertical lines (respectively with horizontal lines), larger deletions occurring within a single *MdAGG* sequence and leading to frameshift mutations are represented with a smaller box with horizontal lines, deletions occurring between different *MdAGG* sequences and leading to large genomic DNA excisions are represented with a two-coloured box, and sequences for which no information is available are represented by light colored boxes. The asterisk (*) corresponds to a specific case observed in clone 4 of *mdagg-4*, where a 200-nucleotide portion of *MdAGG15* was inserted in inverse orientation into the targeted site of *MdAGG3*. gRNA efficiencies are represented in (i) blue if the edits occurred in the gRNA1 targeted sequence, in (ii) red if edits occurred in the gRNA2 targeted sequence and in (iii) green if edits occurred in both gRNA targeted sequences. The targeted phase of the ‘Gala’ genome is specified when possible: orange for phase A, light blue for phase B and black for phase A or B.

A total of 1150 leaf explants were inoculated with *A. tumefaciens* containing the binary vector with the *pPPO16::MdAGG10* construct (T-DNA in Supplementary Data Fig. S1b) assumed to induce *MdAGG10* expression upon *Ea* infection [28]. The nine kanamycin-resistant regenerants that survived over 6 months of micropropagation were analyzed by PCR, with similar controls as described above for CRISPR lines, to confirm transformation and absence of *A. tumefaciens* contamination (Supplementary Data Fig. S2b). Amplification with primers H (Supplementary Data Table S1) for the presence of *pPPO16::MdAGG10* association showed that all lines had integrated the full construct. Considering the initial number of explants (1150), the transformation rate was therefore 0.78%. Six of the nine lines, called *pPPO16::MdAGG10-1* to *pPPO16::MdAGG10-6*, were further analyzed.

### Dual guide-RNA edition of *MdAGG* genes leads to multiple loss-of-function mutations

For each of the five lines, named *mdagg-1* to *mdagg-5*, four bacterial clones (referred to as ‘clones’) were sequenced as a check of the occurrence of editing events (Fig. 1). The two target sites proved effective, and the five lines proved to be edited, displaying various types of edits: (i) indel or substitution of several nucleotides in one *MdAGG* sequence (called hereafter type A edit), (ii) deletion of the sequence between the two target sites, of around 200 nucleotides, in one *MdAGG* sequence (called hereafter type B edit) and (iii) deletion of several thousands of nucleotides between two different *MdAGG* sequences (called hereafter type C edit). This characterization also reported the insertion of one *MdAGG* sequence between the two targeted sites of another *MdAGG* sequence (called hereafter type D edit). In the *mdagg-1* line we observed four type A edits. In the *mdagg-2* line we observed two type B edits (clone 1 and 2), one type A edit (clone 3) and one type C edit (clone 4) displaying a 126-kb deletion. In the *mdagg-3* line we observed three unedited *MdAGG* sequences (clones 2, 3 and 4) and one type A edit (clone 1). For the *mdagg-4* line, clones 1 and 2 displayed type A edits and clone 3 displayed a type C edit with a 212-kb deletion. Ultimately, clone 4 displayed a type D edit, where 200 nucleotides of *MdAGG15* were inserted in inverted orientation between the two targeted sites of *MdAGG3*. For the *mdagg-5* line, we observed three type B edits (clones 1, 2 and 3), as well as one type A edit (clone 4). All these edits on the *MdAGG* coding sequence led to the early insertion of a stop codon resulting in a truncated protein, except for one *MdAGG10* allele (clone 3) of the *mdagg-1* line, where edits led to substitution or indels of amino acids (Fig. 1). Altogether, this analysis showed that both gRNAs were effective and that they led to multiple loss-of-function mutations in the *MdAGG* genes.

### MdAGG loss-of-function mutants are impaired in their ASM-induced resistance response toward *E. amylovora*

**Figure 2:**
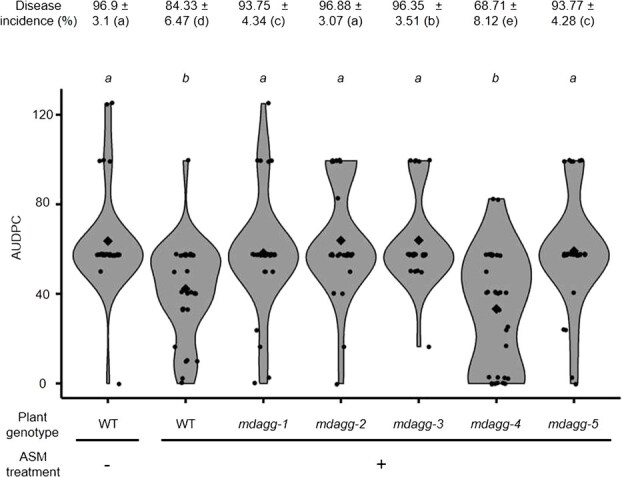
*In vitro* culture shoots of *MdAGG-*edited apple mutants except *mdagg-4* are still susceptible to *Erwinia amylovora* despite ASM treatment. Fire blight disease incidence (± standard deviation) and AUDPC scoring over an 8-day period after *Ea* infection. Each point represents the AUDPC value for individual shoots (28 < *n* < 32); diamonds represent the mean AUDPC for each condition. Significance was evaluated with a Kruskal–Wallis test followed by a Dunn test; different letters indicate different statistical classes (*P* < 0.05).

To assess the effect of loss-of-function mutations of *MdAGGs* in inducible-resistance, *in vitro* shoot cultures of lines *mdagg-1* to *5* were treated with ASM and further inoculated with *Ea*. Susceptible control (untransformed susceptible variety ‘Gala’ (wild-type, WT) treated with water), displayed significantly higher disease incidence and severity scores than resistant one (ASM-treated WT). Compared with the resistant control, fire blight incidence was significantly higher in all *mdagg* mutant lines treated with ASM, except for line *mdagg-4* (Fig. 2). Regarding fire blight severity, the area under the disease progression curve (AUDPC) of ASM-treated *mdagg-4* mutants was equivalent to that of the resistant control whereas the AUDPC of the four other ASM-treated *mdagg* mutants was equivalent to that of the susceptible control (Fig. 2). Altogether, these results show that, except for line *mdagg-4*, *in vitro* shoots of *mdagg* mutant lines were not able to mount a fully effective resistance response to *Ea* upon ASM treatment. Mutant lines *mdagg-1* and *mdagg-2* were further analyzed.

**Figure 3:**
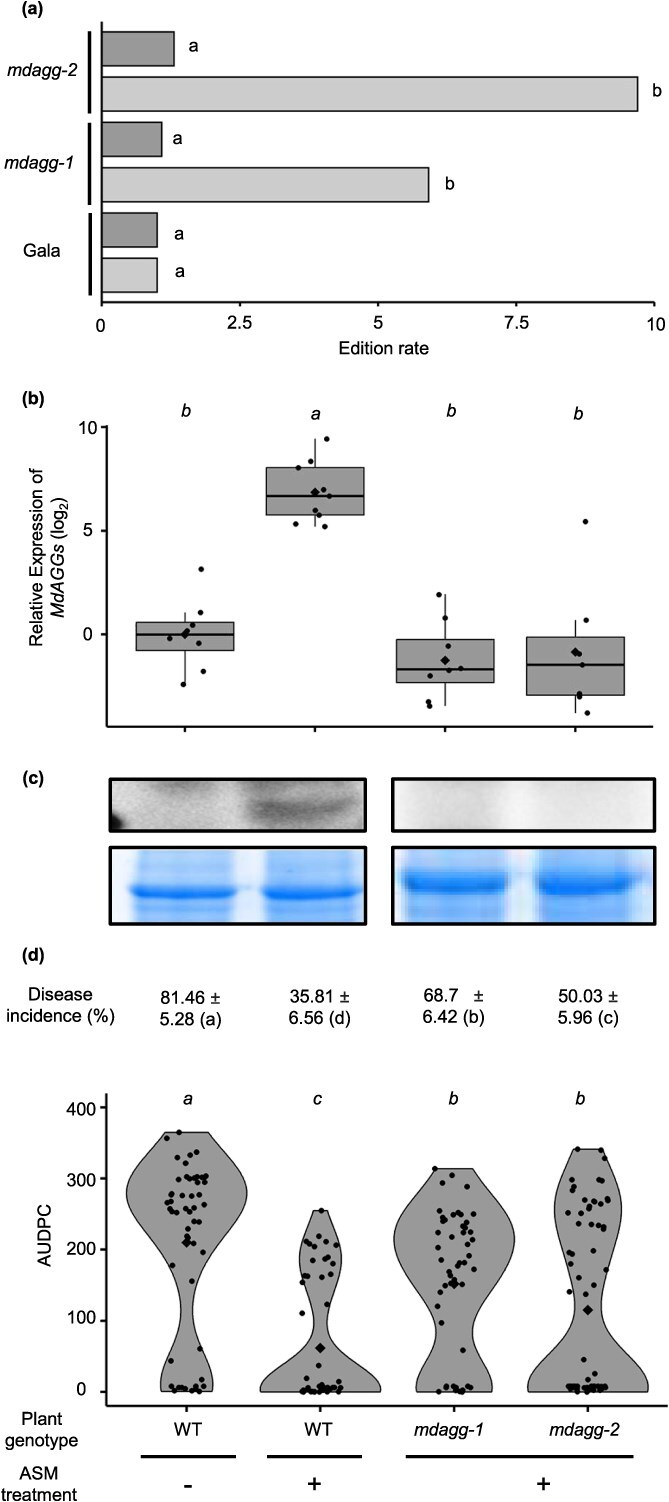
Greenhouse-grown *MdAGG-*edited apple mutants do not upregulate *MdAGG* upon ASM treatment and are more susceptible to *Erwinia amylovora*. (**a**) Quantification of editing rates at the gRNA1 target sequences in *mdagg-1* and -*2* lines. The editing rate (*e*) was calculated as follows: *e* = 1/*x* where *x* corresponds to the relative quantity of unedited DNA sequences of *MdAGG*s (light gray bars) or *MdDiAGG4* (dark gray bars) measured by qPCR. The values shown are the average measured in four to six samples from two independent biological repeats. The data are compared with the ‘Gala’ non-transformed genotype as an unedited reference. Significance of the Wilcoxon rank test: different letters indicating significant differences (*P* < 0.05). (**b**) Relative expression (log_2_ ratio) of *MdAGGs* in the non-inoculated leaves just before bacterial infection. Means, medians and interquartile range (IQR) are indicated by diamonds, lines and boxes, respectively. Whiskers indicate the most extreme values between the nearest quartile and 1.5 IQR. The mean value from samples of water-pretreated controls (WT) was used for the calculation of the log_2_ ratio. Each dot represents one biological replicate, i.e. a pool of two leaves of independent plants (7 < *n* < 9). (**c**) MdAGG protein detection by western blot (upper panel) in non-inoculated leaves just before bacterial infection. Homogeneity of loaded proteins (15 μg per lane) was verified by Coomassie brilliant blue staining (lower panel). (**d**) Fire blight disease incidence (± standard deviation) 21 days after *E. amylovora* infection and phenotypic assessment by AUDPC calculation over these 21 days. Each point represents the AUDPC for individual plants (52 < *n* < 70 from two independent biological repeats). Diamonds represent the mean AUDPC for each condition. For all quantified parameters, significance was evaluated with a Kruskal–Wallis test followed by a Dunn test, different letters indicating significant differences (*P* < 0.05).

We completed the quantitative characterization of the edits in *mdagg-1* and *mdagg-2* using a qPCR-based method we developed to measure the editing rates at the gRNA1 targets, because this guide seemed to be very effective for MdAGG edits (Fig. 1). The targets of gRNA1 consisted of MdAGG target sites and the MdDiAGG4 off-target site (Table 1). The forward primers for both the MdAGG targets and MdDiAGG4 off-target were designed to end just after the Cas9 cut site (four nucleotides from the PAM) of the gRNA1 target. This design allows amplification only in wild-type alleles, where no cutting or modification has occurred, and in rare cases where synonymous repairs may have taken place. Estimated global editing rates were then calculated as inverses of these quantities of unmodified sequences. The results show that both mutant lines *mdagg-1* and *-2* were significantly edited at the *MdAGG* target sites (Fig. 3a, light gray bars), while neither displayed significant edits at the off-target site *MdDiAGG4* (Fig. 3a, dark gray bars).

Next, to determine if *MdAGG*s are involved in the ability of greenhouse-grown apple to mount an effective ASM-induced resistance response to *Ea*, mutant lines *mdagg-1* and *mdagg-2* were acclimatized in a greenhouse, treated with ASM and further inoculated with *Ea*. WT plants treated with either water or ASM were included as susceptible and resistant controls, respectively. Targeted gene expression and protein production were also analyzed in the remaining leaf tissues cut out during the inoculation procedure. *MdAGG* expression was monitored by RT–qPCR with primers O (Supplementary Data Table S1) designed to allow amplification of transcripts only in the absence of edition (Supplementary Data Fig. S1c). As expected, the results show that *MdAGG* expression was significantly upregulated by the ASM treatment compared with the water treatment in untransformed control plants. Interestingly, no significant difference in *MdAGG* transcript accumulation was observed between the susceptible control and the ASM-treated mutant lines (Fig. 3b), suggesting that among the *MdAGG*s successfully disrupted in these mutant lines are those primarily responsible for the ASM-induced expression observed in the resistant control. Protein accumulation analysis by western blot confirmed loss of function for *mdagg-1* and *mdagg-2* mutants as MdAGG proteins were only detected in the resistant control (Fig. 3c). After inoculation with *Ea*, fire blight disease symptoms were scored every 3–4 days for 3 weeks and resistance was evaluated by determining disease incidence at the end of the experiment or disease severity by AUDPC calculation (Fig. 3d, Supplementary Data Table S2). As expected, the ASM treatment reduced by >50% disease incidence and severity for the WT. In contrast, although disease incidence and severity in ASM-treated *mdagg-1* and -*2* lines were both significantly lower than for the susceptible control, they were significantly higher than for the resistant control (Fig. 3d). This shows that, despite ASM elicitation, *mdagg-1* and *mdagg-2* greenhouse-grown mutants are less resistant to fire blight than the resistant control.

Overall, these results demonstrate that the expression and accumulation of functional MdAGGs are crucial for apple to mount a fully effective resistance response induced by ASM.

### Molecular and phenotyping characterization of *pPPO16::MdAGG10*-inducible lines under *E. amylovora* inoculation challenge

**Figure 4:**
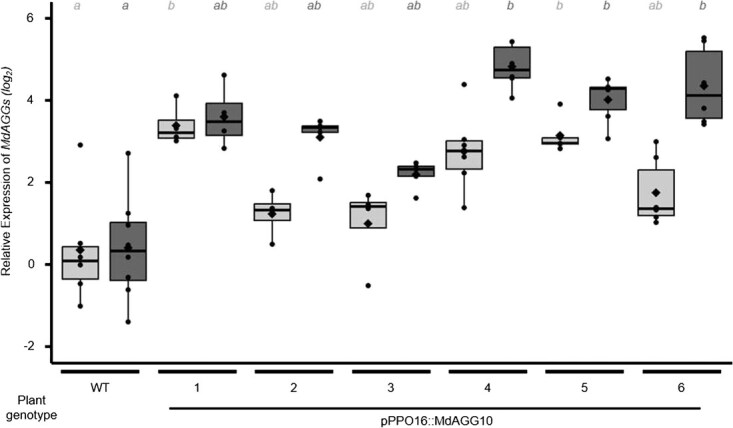
Fire blight-inducible *PPO16* promoter upregulates *MdAGG* expression upon *Erwinia amylovora* infection on *in vitro* culture shoots of *pPPO16::MdAGG10* transgenic lines. Light (respectively dark) gray boxplots represent *MdAGG* expressions 24 (respectively 40) h after *E. amylovora* infection by infiltration, versus water infiltration (mock). Each point represents the *MdAGG* expression (log_2_ ratio) in individual inoculated samples relative to the mean value of *MdAGG* expressions in the water-infiltrated biological repeats at the same day of sampling. Means, medians and interquartile range (IQR) are indicated by diamonds, lines and boxes, respectively. Each biological replicate is a pool of all leaves sampled from two independent plants (6 < *n* < 8). Significance was evaluated with a Kruskal–Wallis test followed by a Dunn test; different letters indicate different significance (*P* < 0.05). Boxes with the same letter and the same color represent medians that are not significantly different (*P* < 0.05).

Six of the nine lines obtained after *A. tumefaciens* transformation, called *pPPO16::MdAGG10-1* to *pPPO16::MdAGG10-6*, were analyzed to determine if bacterial infection with *Ea*, known to activate the *pPPO16* promoter, effectively led to overexpression of *MdAGG10*. Transcript accumulation of *MdAGG10* was assessed by RT–qPCR at 24 and 40 h post-infiltration (hpi) in mock- or *Ea*-infiltrated leaves of *in vitro* shoot cultures. The untransformed ‘Gala’ variety (WT) was used as a control. Differential gene expression analysis shows that untransformed plants did not upregulate *MdAGG10* in response to *Ea* infection whereas the transformed lines did, with a log_2_ ratio (compared with the WT line) varying from 1 to 5 depending on the line and the day of sampling (24 or 40 hpi; Fig. 4).

**Figure 5:**
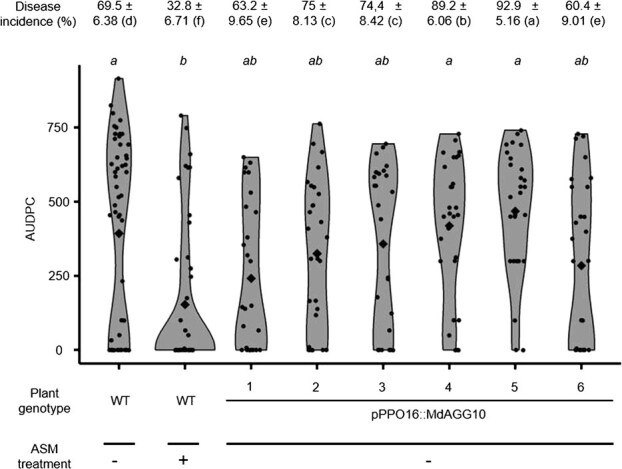
*In vitro* culture shoots of *pPPO16::MdAGG10* lines are less susceptible to *Erwinia amylovora.* Fire blight disease incidence (± standard deviation) 12 days after *E. amylovora* infection and phenotypic assessment by AUDPC calculation over these 12 days. Each dot represents the AUDPC of one shoot (29 < *n* < 35). Each diamond represents the mean AUDPC of each condition. Significance was evaluated with a Kruskal–Wallis test followed by a Dunn test; different letters indicate different statistical classes (*P* < 0.05).

To determine if conditional upregulation of *MdAGG10* enhances apple resistance to *Ea*, *in vitro* shoot cultures of *pPPO16::MdAGG10-1* to -*6* lines were inoculated with *Ea* and disease symptoms were scored daily over 12 days following inoculation. Resistance was evaluated by determining disease incidence at the end of the experiment or disease severity by AUDPC calculation. The WT plants, treated either with water or ASM, were included as respectively susceptible and resistant controls. Water-treated WT plants displayed significantly higher disease incidence and severity scores than ASM-treated WT plants. Fire blight incidence was significantly higher than in the susceptible control in four of the six lines (*pPPO16::MdAGG10-2* to -*5*), and intermediate to those of resistant and susceptible controls in *pPPO16::MdAGG10-1* and -*6*, although more similar to incidence measured for the susceptible control (Fig. 5). In four of the six lines (*pPPO16::MdAGG10-1* to -*3* and -*6*), disease severity was intermediate to those of resistant and susceptible controls, whereas in *pPPO16::MdAGG10-4* and -*5*, it was equivalent to that of the susceptible control (Fig. 5). In brief, except lines *pPPO16::MdAGG10-4* and -*5*, most of the *in vitro* shoots of *pPPO16::MdAGG10* lines were less severely affected by fire blight than the susceptible control. Lines *pPPO16::MdAGG10-1* and *pPPO16::MdAGG10-6* were further analyzed in a greenhouse.

**Figure 6:**
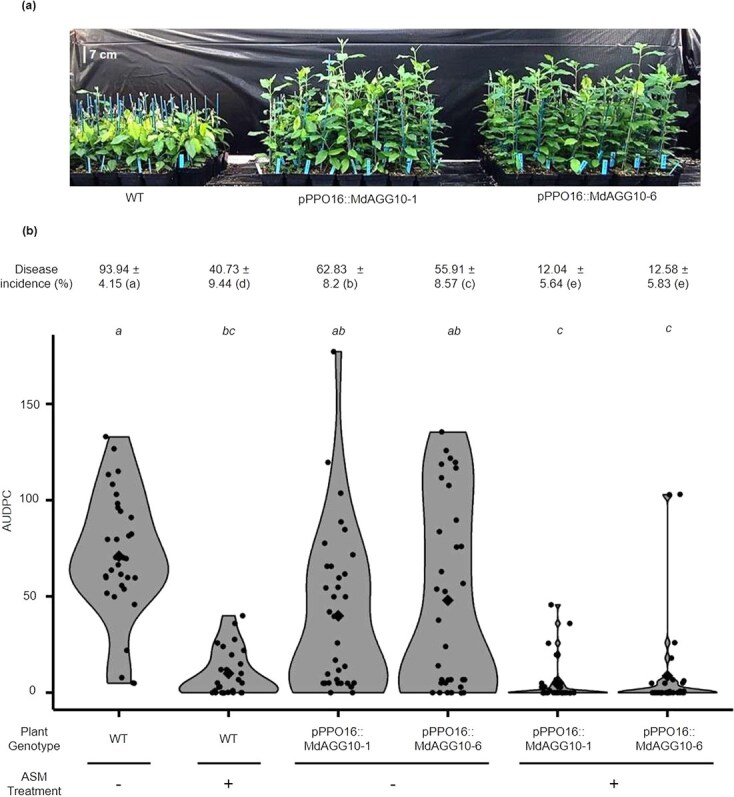
Greenhouse-grown *pPPO16::MdAGG10* apple lines are less susceptible to *Erwinia amylovora*. (**a**) Representative picture of water-treated apple lines inoculated with *E. amylovora*, 19 days post-inoculation. (**b**) Fire blight disease incidence (± standard deviation) 19 days after inoculation and phenotypic assessment by AUDPC calculation over these 19 days. Each dot represents the AUDPC of one plant (29 < *n* < 35). Each diamond represents the mean AUDPC of each condition. Significance was evaluated with a Kruskal–Wallis test followed by a Dunn test; different letters indicate different statistical classes (*P* < 0.05).

We next investigated whether infection-inducible *MdAGG10* expression provides effective resistance to *Ea* lines *pPPO16::MdAGG10-1* and -*6* grown in a greenhouse with no apparent development problems (Supplementary Data Table S3), and if ASM further enhances this resistance, knowing that the *pPPO16* promoter had little reaction to ASM, in comparison with its reaction to *Ea* infection (Supplementary Data Fig. S3, adapted from [21]). Plants were therefore treated with ASM or water and then inoculated with *Ea*. The untransformed susceptible variety ‘Gala’, treated with either water or ASM, was included as a respectively susceptible and resistant control. Nineteen days after inoculation, plants of water-treated *pPPO16::MdAGG10-1* and -*6* lines were obviously less affected by fire blight compared with the susceptible control. Disease incidence reached almost 94% in the susceptible control, which results in the stunted phenotype of plants systemically affected by *Ea* (Fig. 6a). In contrast, significantly lower disease incidence of the water-treated *pPPO16::MdAGG10-1* and -*6* plants is evidenced by actively growing plants for both of these transgenic lines (Fig. 6a and b). Regarding disease severity, water-treated *pPPO16::MdAGG10-1* and -*6* lines were intermediate to those of resistant and susceptible controls. Plants of both transgenic lines that were previously treated with ASM displayed lower disease incidence and severity than the resistant control (Fig. 6b, Supplementary Data Table S4).

Altogether, these results show that infection-induced *MdAGG10* expression alone provides effective resistance to *Ea* in greenhouse-grown plants, with ASM treatment offering additional protection, reducing disease symptoms by ~90%.

## Discussion

Genetic improvement by NBT of apple varieties susceptible to fire blight has already been performed by transferring QTL genes from wild relatives, or knocking-out of S genes. Here, we provide evidence that intragenic association of a disease-induced promoter with a downstream defense gene constitutes a supplementary strategy for the biotechnological toolbox aiming at enhancing and studying fire blight disease resistance in apple.

From a methodological point of view, being able to use apple shoots cultivated *in vitro* for pre-screening of transformed lines, before their acclimatization in a greenhouse, can save valuable time and money. In this work, although the observed effects were weak, they nonetheless allowed pre-screening, the results of which were then confirmed and reinforced in a greenhouse for the two selected lines, as already observed for plum resistance to sharka [31], but only partially for pear resistance to fire blight, presumably due to a higher inoculum concentration in the greenhouse than *in vitro* [32].

Still, from a methodological point of view, the two gRNAs designed for targeting all *MdAGG* members of the family and the tandem array organization of *MdAGG1* to *MdAGG15* on chromosome 10 [22] allowed large DNA deletions of several kilobases eliminating numerous *MdAGG* sequences in their entirety. CRISPR/Cas9 is a powerful and precise method to induce targeted mutagenesis, but it is known to create editing chimerism in plants whose transformation involves somatic organogenesis potentially involving several cells at the origin of the regenerated line, which is the case for apple [29]. Rounds of adventitious regeneration steps can efficiently reduce chimerism [33] but stay time-consuming. As already demonstrated by Pompili *et al.* [19], high-throughput sequencing (HTS) targeted resequencing methods can be used to capture this edition diversity in first-generation edited apple lines, both in qualitative and quantitative terms. As an initial strategy, we opted here for a partial qualitative sequencing analysis of the edits by examining a few alleles of the targeted sequences. This strategy was intended to verify that editing events had occurred, but it could not provide the complete editing profile of each line. This approach nevertheless provided a solid overview of the different types of edits involved, especially the large deletions. In the mutant lines *mdagg-1* and -*2*, selected for further study, the gRNA1 targets consisting of *MdAGG* target sites and the *MdDiAGG4* off-target site were then analyzed quantitatively using an easy-to-use and effective qPCR-based method, confirming that these lines were suitable for robust greenhouse experiments.

We first demonstrated *in vitro* that four of the five *mdagg* lines were more susceptible to fire blight than the WT plants despite the application of ASM. Mutant *mdagg-4* was the only line whose ASM-induced resistance was comparable to that of WT plants, although it displayed editions affecting *MdAGG* sequences. It is unclear whether cell types that are at the first line in the early steps of the foliar infection process, such as those surrounding the vascular tissue [34], remained unedited for this mutant, or if certain edits, such as the 212-kb deletion observed in one clone, enhance resistance by eliminating unidentified susceptibility factors. An in-depth analysis of this mutant line remains to be performed to explain its inconsistent phenotype. For the mutants selected for greenhouse assays, the decrease in resistance to *Ea* correlated with the absence of MdAGG accumulation at the transcriptional and protein levels. To the best of our knowledge, this is the first time that amaranthin-like lectins have been demonstrated to be involved in plant resistance against a bacterium by loss of function in the native species rather than through ectopic expression [35]. Here, MdAGGs are lectins containing a unique amaranthin-like domain, able to agglutinate bacterial cells *in vitro* but devoid of proven bactericidal effect [27]. Whether MdAGGs act by physically slowing bacterial progression within apple tissues thanks to their agglutination properties or if this agglutination also facilitates the antimicrobial activity of other plant-derived compounds remains to be investigated.

Once we had demonstrated that MdAGGs are necessary to mount an effective ASM-triggered resistance response to fire blight, we wanted to determine if gain of function by inducible expression of MdAGG10 improves resistance to *Ea*. Because of its high expression after ASM treatment and its closest proximity to the consensus sequence of the other 17 members of the family [22], we chose *MdAGG10* as the best candidate to select, to promote fire blight resistance. A previous attempt performed by constitutive overexpression of *MdAGG10* did not meet our expectations insofar as overexpressing lines were as susceptible to *Ea* as wild-type ones [27]. We wondered whether plants engineered to conditionally express *MdAGG10* following *Ea* infection could display partial resistance to fire blight. The *Ea*-inducible promoter *pPPO16* [28] was therefore used to produce transgenic *pPPO16::MdAGG10* apple lines. Here, our results showed that *MdAGG10* exhibited strong expression in the early stages of *Ea* infection, thanks to its association with *pPPO16*, and this strong expression was associated with enhanced resistant to the disease. Moreover, combining inducible expression of *MdAGG10* and treatment with ASM led to a drastic reduction of the disease. The *pPPO16* promoter being much less responsive to ASM treatment than to *Ea* infection, it is rather unlikely that this additional disease reduction is due to the small additional induction of the promoter by ASM. The mechanisms responsible for the important disease reduction evidenced in ASM-treated *pPPO16::MdAGG10* lines remain to be determined. These plants could either have benefited from the addition of *Ea*-induced *MdAGG10* cisgene expression with that of the ASM-induced *MdAGG* endogenes, but they could also have been protected thanks to the induction of hundreds of other defense-related genes known to be upregulated by ASM [22]. These results, when compared with those from the previous study involving constitutive overexpression of the same *MdAGG10* gene [27], underscore the advantage of prioritizing inducible expression over constitutive expression when the aim is to enhance the activity of genes of interest. Inducible and transient expression of *MdAGG10* here had no negative impact on plant development and conferred partial resistance, whereas its constitutive CaMV p35S-driven overexpression led to pleiotropic effects on plant growth without enhancing resistance [27]. Such pleiotropic effects have already been documented in studies dedicated to constitutive overexpression of lectin-encoding genes [35]. The past studies that demonstrated that the ectopic expression of the amaranthin-encoding gene from *Amaranthus caudatus* protects recipient plants from aphids without pleiotropic effects were performed using phloem-specific promoters [36]. It seems therefore that amaranthin-like-encoding genes can be engineered to promote plant resistance but their regulation in space and time has to be considered cautiously to avoid adverse effects on plant fitness. Using native pathogen- or pest-inducible promoters like *pPPO16* to drive amaranthin-like lectin-encoding genes, as we did here, could be an interesting option to test in other systems.

Until now, breeding apple resistance has focused on resistance QTLs [14]. However, the ongoing plant–pest arms race often results in the selection of mutations in the pest’s genome. These mutations alter the structure of its avirulence proteins, allowing it to bypass the plant’s R genes and evade recognition. Bypasses of resistance QTLs to *Ea* have already been observed in apple [12]. Here, we demonstrate that it is possible to create a resistance in apple by focusing on a downstream defense gene, *MdAGG10*, which interacts with *Ea*’s PAMPs LPS and EPS, possibly through electrostatic interactions [21]. Indeed, the most important feature seems to be the acidic (i.e. negatively charged) nature of the polysaccharide [21]. Mutants of *Ea* engineered to produce uncharged amylovoran (the principal component of *Ea*’s EPS) were shown to be non-pathogenic on apple, phenocopying bacterial mutants that do not produce amylovoran at all [37]. Altogether, these studies suggest that the downstream defense protein MdAGG10 targets bacterial features under strong evolutionary constraints, possibly making it difficult for *Ea* to bypass the provided resistance. These considerations lead us to believe that the intragenic *pPPO16–MdAGG10* association is a good candidate for breeding durable resistance.

### Conclusions

In this work we have validated MdAGGs as important compounds of apple’s defense toolbox against *Ea* and created a partial resistance to fire blight thanks to the intragenic association of the disease-inducible *pPPO16* promoter and the coding sequence of *MdAGG10*. But the *pPPO16::MdAGG10* plants analyzed here do not meet the standards of intragenic plants: (i) the terminator of this construct is of viral origin and (ii) the corresponding T-DNA does not have a recombination system such as in the PMF1-GFP-dest plasmid [38], which allows the elimination of other elements (selection gene, recombination system itself) than the construct of interest after transformation. Functional intragenic apple lines could be obtained using the Rubisco terminator, which has already been employed in apple [17]. Combined with the above-mentioned recombination system, such intragenic lines allowing *MdAGG10* expression upon *Ea* infection could be upgraded towards higher resistance by pyramiding more genes involved in partial resistance. Further combined with other control methods, such as treatments with environment-friendly non-chemical plant resistance inducers, unlike ASM, they will contribute to generate robust and high-level resistance enabling better control of fire blight.

## Materials and methods

### Bacterial strains, culture and suspension preparation

*Agrobacterium tumefaciens* strain EHA105 containing plasmids of interest was grown in Luria Broth (LB) medium (Duchefa Biochemie, DH Haarlem, Netherlands) at 28°C. Suspensions of *A. tumefaciens* were prepared in a medium consisting of 1.95 g/l MES medium (Duchefa Biochemie), 2 g/l MgCL_2_, 20 g/l sucrose and 30 mg/l acetosyringone, with pH adjusted to 5.6. *Ea* strain CFBP1430 was grown on King’s B medium [39] at 26°C. Suspensions of *Ea* were prepared in sterile water from solid exponential growth phase culture.

### Construction of vectors and generation of transgenic lines

The binary vector CRISPR-MdAGGAll used in this study (T-DNA in Supplementary Data Fig. S1a) was derived from pDE-CAS9Kr vector [29]. To maximize the probability of targeting the 18 *MdAGG* genes, this construct contained two guide RNAs with a different promoter (gRNA1 with MdU3 and gRNA2 with MdU6), which were designed so that either one or both are complementary to each *MdAGG* gene while minimizing off-target sequences (Table 1) *MdDiAGG4* was the only coding sequence potentially off-targeted by gRNA1 and ABS1 was not considered because it is non-coding and there is no evidence of transcription factor binding sites within it (checked on PlantPAN https://plantpan.itps.ncku.edu.tw/plantpan4/TF_TFBS_search.php with *Arabidopsis* and *Malus domestica* genomes [40]). We speculated that this strategy would lead to (i) point mutations at either gRNA site, (ii) deletions of several hundreds of nucleotides between both gRNA sites within *MdAGG* genes or (iii) deletions of several thousands of nucleotides between gRNA sites of different *MdAGG* genes organized in tandem array. Each gRNA cassette flanked with attB gateway sites was synthesized independently by Integrated DNA Technology, Inc. (San Jose, CA, USA) and subsequently cloned in pDONR207 vector via BP cloning (Gateway system; Thermo Fisher Scientific, Waltham, MA, USA). The vector pDONR207-U3gRNA1-U6gRNA2 was constructed by placing the U6gRNA2 cassette after the U3gRNA1 cassette using restriction/ligation at XhoI/PstI sites in the donor vector followed by SalI/PstI cloning in the destination vector. It was then used to create vector CRISPR-MdAGGAll using Gateway LR cloning with the vector pDE-CAS9Kr. Primers used to verify each cloning step (primers A: BP cloning and gRNAs addition; primers B: LR cloning) are indicated in Supplementary Data Table S1. BLAST analysis with AtU3 and AtU6 sequences (respectively, found upstream of X52629 and X52528 [41]) confirmed that MdU3 and MdU6 promoters are respectively placed upstream of MD10G1073100 and MD07G1138500 genes (https://iris.angers.inra.fr/gddh13/). Sequences are given in Charrier *et al.* [29]. The CRISPOR software (http://crispor.tefor.net/ [42]) was used to choose the target sequences of gRNAs based on version 1.1 of the *Malus domestica* INRAE GGDH13 genome (https://iris.angers.inra.fr/gddh13/ [43]).

The binary vector pPPO16::MdAGG10 used in this study (T-DNA in Supplementary Data Fig. S1b) was derived from pKGWFS7 [44] and was constructed as follows. The promoter *pPPO16* from ‘MM106’ (MK873007, ~2 kb) was amplified with Q5 High-Fidelity DNA Polymerase (New England Biolabs, Ipswich, MA, USA) according to the manufacturer’s instructions and with primers C adding an EcoRI restriction site at 3′. The amplified fragment was then cloned into p-ENTR/D TOPO (Invitrogen, Carlsbad, CA, USA) and then transferred into the destination vector pKGWFS7 by Gateway recombination to create pKGWFS7-pPPO16. The *MdAGG10* CDS (MD10G1027210, 498 bp) was also amplified with the Q5 high-fidelity DNA polymerase according to the manufacturer’s instructions and with primers F, adding EcoRI and NcoI restriction sites at 5′ and 3′, respectively, then cloned into pGEM-T easy (Promega, Madison, WI, USA) according to the manufacturer’s instructions. The *EcoRI*-MdAGG10-NcoI fragment was then inserted in pKGWFS7-pPPO16 by restriction/ligation to create pPPO16::MdAGG10. Primers used to clone or to verify each cloning step (primers C–H) are indicated in Supplementary Data Table S1.

Apple transgenic lines were generated as previously described in Malabarba *et al.* [33]. Briefly, transformation was performed on the youngest leaves of 4-week-old *in vitro* apple shoots. The leaves were vacuum-infiltrated at −0.9 bar for 1 min in a suspension of *A. tumefaciens* (EHA105) at 1 × 10^8^ bacteria/ml and Silwet L-77R (Lehle Seeds, Round Rock, TX, USA) at 0.002%. Leaves were then wounded transversely with a scalpel and transferred to apple regeneration medium containing Murashige and Skoog salts (MS) [45], 30 g/l saccharose, 5 mg/l thidiazuron, 0.5 mg/l naphthalene acetic acid and 100 mM acetosyringone, solidified with Phytagel™ (Sigma–Aldrich, Saint Louis, MO, USA) at 3 g/l. Leaves were co-incubated for 2 days in the dark at 22–24°C with *A. tumefaciens*. Leaves were then placed on their respective regeneration medium containing 150 mg/l timentin, 100 mg/l kanamycin and 300 mg/L cefotaxime. Explants were kept in dark conditions for a total of 6 months, transferred monthly to fresh medium and monitored for emergence of adventitious buds.

### Plant growth conditions and ASM treatment

*In vitro* shoots of non-transgenic ‘Gala’ were micropropagated as in Malabarba *et al.* [33], on MS salts [45] supplemented with 30 g/l saccharose, 0.1 mg/l 3-indolebutyric acid (IBA) and 0.5 mg/l 6-benzyladenine and solidified with agar (Condalab, Madrid, Spain) at 7.5 g/l. Regenerated buds from transformation and subsequent *in vitro* shoots of transgenic lines were micropropagated in the same medium, with the addition of 150 mg/l timentin, 100 mg/l kanamycin and 300 mg/l cefotaxime. Cultures were transferred monthly to fresh medium. All the plants were cultivated at 22–24°C in a growth chamber with a 16:8 h light:dark photoperiod (cool white fluorescent tubes, 40–60 mmol m^−2^ s^−1^).

The rooting conditions were as follows: *in vitro* shoots of non-transgenic ‘Gala’ and of transgenic lines were placed for 1 week in the dark and at 22–24°C, on an induction medium composed of MS salts [45] supplemented with 20 g/l saccharose and 3 mg/l IBA and solidified with agar (Condalab, Madrid, Spain) at 7.5 g/l. *In vitro* shoots were then placed for 3 weeks, at 22–24°C with a 16:8 h light:dark photoperiod, on an expression medium composed of MS salts [45] supplemented with 20 g/l saccharose and solidified with agar (Condalab) at 7.5 g/l. The rooted shoots were then acclimatized into a greenhouse and grown under 22°C, humidity rate of 80% and shading of 500 W m^−2^ for 8 weeks after acclimation. These 8-week-old acclimatized plants are referred to as ‘greenhouse-grown plants’ throughout the manuscript, whether they are non-transgenic ‘Gala’ control plants or transgenic lines.

*In vitro* shoots and greenhouse-grown plants (non-transgenic control and transgenic lines) were treated with a 0.2 g l^−1^ ASM (Bion^®^ 50WG, Syngenta, Basel, Switzerland) solution 3 days before inoculation, with either an Ecospray sprayer (Seidden, Madrid, Spain) for *in vitro* shoots or a 750-ml hand sprayer for greenhouse-grown plants. The ASM solution was filter-sterilized at 0.22 μm (Pall Corporation, Port Washington, NY, USA) before spraying the *in vitro* shoots.

### Plant inoculation, phenotyping and sampling

For phenotyping analyses (Figs 2, 3d, 5 and 6b), WT or transgenic *in vitro* shoots and greenhouse-grown plants were inoculated with a suspension of 10^7^ colony forming units mL^−1^ of *Ea* prepared in sterile water. Plants were inoculated with scissors previously dipped in *Ea* suspension, by cutting the lower third of one of the apical leaf for *in vitro* shoots, or of the last fully developed leaf for greenhouse-grown plants. In the experiments performed on WT or *mdagg-1* and -*2* lines grown in the greenhouse (Fig. 3b and c), the remaining leaf tissues cut out for inoculation were then pooled in pairs and frozen at −80°C for subsequent analysis of *MdAGG*s expression (7 < *n* < 9) or MdAGGs protein detection (*n* = 1). Fire blight incidence was measured by checking the presence of necrotized cells beyond the inoculated leaf petiole. Fire blight severity corresponded to the area under the disease progression curve (AUDPC [46]) calculated with fire blight symptoms measured differently on *in vitro* shoots or greenhouse-grown plants. On greenhouse-grown plants, they were measured 4, 7, 11, 14 and 19 days post-inoculation (dpi) by measuring the length of necrotized shoot, whereas on *in vitro* shoots a score was assigned at 4, 5, 6, 7 and 8 dpi for the *MdAGG-*edited lines test (Fig. 2) and at 4, 5, 6, 7, 8, 10 and 12 dpi for the *pPPO16::MdAGG10* lines test (Fig. 5), according to the proportion of necrotized tissues observed on each plant. These scores are summarized in Supplementary Data Table S5. For expression analyses on *in vitro* shoots of non-transgenic ‘Gala’ control and *pPPO16::MdAGG10* transgenic lines 1–6 (Fig. 4), inoculation was done similarly as in Gaucher *et al.* [28]. Bacterial inocula were prepared in sterile water at a concentration of 10^7^ colony forming units ml ^−1^, supplemented with 0.01% (v/v) of wetting agent Silwet (L-77, De Sangosse Ltd, Cambridge, UK). The mock solution corresponded to sterile water supplemented with the wetting agent Silwet. Shoots were totally submerged in mock or inoculum and vacuum-infiltrated at −0.09 Mp for 2 min. Infiltrated shoots were dried on sterile filter paper and placed on micropropagation medium for 3 days before sampling. Twenty-four and 4 hpi, mock- or *Ea*-infiltrated shoot leaves were sampled, immediately frozen in liquid nitrogen and kept at −80°C until analysis. Each biological replicate is a pool of all leaves sampled from two independent shoots (6 < *n* < 8).

### Nucleic acid extraction and reverse transcription

Genomic DNA (gDNA) was extracted as described in Fulton *et al.* [47]. For RNA extraction, frozen leaf samples were ground to powder using a tissue lyser (Retsch, Hann, Germany) for 20 s at 25 Hz twice. Total RNA was extracted with a NucleoSpin RNA Plant Kit (Macherey-Nagel, Düren, Germany) following the manufacturer’s recommendation and RNA concentration was measured with a Nanodrop spectrophotometer (Thermo Fisher Scientific). Two micrograms of RNA was reverse-transcribed into cDNA using M-MLV Reverse Transcriptase (Promega) following the manufacturer’s instructions. Primers I (Supplementary Data Table S1), designed from either side of an intron, were used to confirm the absence of genomic DNA by PCR.

### Genotyping of transgenic lines by PCR and targeted DNA sequencing

T-DNA insertion verifications with primers B, H, I, J, K and L (Supplementary Data Table S1) were performed by PCR amplification with GoTaq G2 Flexi DNA polymerase (Promega) according to the manufacturer’s instructions and by using the following PCR reaction conditions: 95°C for 5 min; 35 cycles at 95°C for 30 s, 58°C for 45 s and 72°C for 60 s, followed by a final extension step at 72°C for 5 min. PCR products were separated on a 2% agarose gel.

For the qualitative characterization of MdAGGs edits, we used primers M (Supplementary Data Table S1) flanking the sequence containing the target zones of the two gRNAs. Amplifications were performed with Q5^®^ High Fidelity 2X MasterMix according to the manufacturer’s recommendations. The PCR reaction was performed with an initial step at 98°C for 30 s followed by 40 cycles at 98°C for 10 s, the appropriate melting temperature for 30 s and 72°C for 60 s and a final extension at 72°C for 120 s. Amplicons were cloned with a CloneJET PCR kit (Thermo Fisher Scientific) according to the manufacturer’s recommendations, and bacterial transformation was performed with Top10 competent *E. coli* cells subsequently spread on LB medium complemented with 100 mg ml^−1^ ampicillin for 24 h at 37°C. Bacterial clones, each containing one allele of the potentially edited *MdAGG* target sequence, were selected by on-colony PCR using N primers (Supplementary Data Table S1) present on the plasmid and framing the insertion zone, with GoTaq G2 Flexi DNA polymerase according to the manufacturer’s recommendations. Selection was on the basis of the expected size of the amplicons inserted in the event of slight modifications of the gRNA targeted sequences (indels or substitutions) or of elimination of the portion of sequence between the two gRNA targeted sequences. For each transgenic line, four bacterial clones containing putative edited MdAGG sequences were sent for sequencing with the reverse N primer (Supplementary Data Table S1) to Azenta Life Sciences (Burlington, MA, USA). The resulting sequences were aligned with the CDS of *MdAGG1* to *MdAGG18* from the two phases of the ‘Gala’ genome [30] using MultAlin software [48].

### qPCR analysis

*MdAGG* expressions and quantifications of *MdAGGs* and *MdDiAGG4* edition rates in *mdagg-1* and *mdagg-2* lines were measured by qPCR. Briefly, cDNA (expression) or genomic DNA (edition rate) was mixed with MESA Blue 2X PCR MasterMix for SYBR Green Assays with fluorescein (Eurogentec, Liege, Belgium) and primers (O for expressions, P and Q for edition rates; Supplementary Data Table S1) at the appropriate concentrations. Reactions were performed on a CFX Connect Real-Time System (Bio-Rad Laboratories, Hercules, CA, USA) for the qPCR and melt curves were produced to check the absence of non-specific amplifications and primer–dimer products. Relative expressions and edition rates quantifications were calculated using the 2-ΔΔCt method. The normalization factor was calculated using three housekeeping genes (*Actin*, *GAPDH* and *TuA*; primers R, S and T, respectively, in Supplementary Data Table S1) as recommended by Vandesompele *et al.* [49].

### Protein extraction, separation and immunodetection

Frozen leaves were ground into powder in liquid nitrogen with a pestle and mortar. Phenol extraction of proteins and subsequent Bradford quantification were performed as described in Chavonet *et al*. [21]. Extracted proteins were separated on Mini-PROTEAN TGX Precast Gel (Bio-Rad Laboratories Inc., Hercules, CA, USA) for 35 min at 200 V (Mini-PROTEAN^®^ Tetra Cell, 4-Gel System, Bio-Rad Laboratories Inc., Hercules, CA, USA) and electroblotted onto 0.45 μm polyvinylidene difluoride (PVDF) membranes (Immobilon-P, Millipore Corp., Bedford, MA, USA). The membrane was subsequently blocked with EveryBlot Blocking Buffer (Bio-Rad Industries Inc., Hercules, CA, USA) and incubated overnight at 4°C in EveryBlot Blocking Buffer containing 1:1000 of two anti-MdAGG antibodies described by Warneys *et al*. [22]. These antibodies are able to recognize all possible MdAGG proteins since they were obtained through rabbit immunization programs (Eurogentec, Liege, Belgium) with synthetic peptides (NKYLRYQLDAESDLN and TEKPNNEDYADKNYV) corresponding to conserved amino acids in MdAGGs. The membrane was then washed five times for 5 min in a TBSt solution (Tris 20 mM; NaCl 150 mM; Tween20 0.005%; v/v; pH 7.6), incubated for 1 h in EveryBlot Blocking Buffer, containing 1:5000 of HRP conjugated goat anti-rabbit antibodies (Merck KGaA, Darmstadt, Germany) and revealed with the Clarity™ Western ECL Substrates Kit (Bio-Rad Laboratories Inc., Hercules, CA, USA) according to the manufacturer’s protocol. Membranes were revealed using a ChemiDoc™ MP Imaging System (Bio-Rad Laboratories Inc., Hercules, CA, USA). Before blotting, the gels were activated under UV light in the same imaging system, which made it possible to reveal total proteins on blotted membranes by fluorescence emission.

### Data analysis

Data analysis was performed using RStudio software [50] and the graphical representations were generated using the package ‘ggplot2’ [51] in association with ‘ggpubr’ [52]. To perform statistical analysis using standard deviations on the single values of fire blight incidence (one value per genotype × condition), we carried out bootstrap analysis with the package ‘boot’ [53]. Tukey’s parametric test (one-step multiple pairwise comparison) was used to assess whether or not the mean sizes for *pPPO16::MdAGG10-1* and -*6* plants were different from those obtained for the non-transgenic control ‘Gala’ (Supplementary Data Table S3), using the package ‘rstatix’ [54]. A non-parametric test (Kruskal–Wallis) followed by a *post hoc* Dunn test was used for all other experiments, using package ‘FSA’ [55] in association with ‘rcompanion’ [56].

## Supplementary Material

Web_Material_uhaf262
