# Supplementary material for: Inducible MdAGG lectins in apple immunity toward fire blight: CRISPR/Cas9 validation and their potential for intragenesis approaches
Source: Hortic Res. 2025 Oct 3;13(1):uhaf262. doi: 10.1093/hr/uhaf262 (PMC12861480; doi:10.1093/hr/uhaf262)
Supplement: Web_Material_uhaf262 [file web_material_uhaf262.zip › SI_Second Review in yellow_VF 20250829.docx]

## Supporting Information

Article title: MdAGG apple lectins in Fire Blight resistance: CRISPR/Cas9 validation and their potential for intragenesis approaches.

Authors: Antoine Bodelot, Nicolas Dousset, Elisa Ravon, Christelle Heintz, Marie-Noelle Brisset, Alexandre Degrave, Emilie Vergne

The following Supporting Information is available for this article:

**Fig. S1** T-DNA constructs and positions of gRNAs targets, quantitative PCR primers, and western-blot probes, on *MdAGG* sequences.

**Fig. S2** Genotyping of apple transgenic lines. (a) PCR on genomic DNA of the five transgenic apple lines with CRISPR/Cas9 targeting *MdAGG* sequences.

**Fig. S3** *MdAGGs* and *MdPPOs* expression modulation by *Ea* *T3SS* in the susceptible MM106 genotype compared with modulation by ASM in apple seedlings (Adapted from Chavonet et *al.*, 2022).

**Table S1** Primer used in this work.

**Table S2** Fire blight lesion length on *mdagg* lines 21 days after *Erwinia amylovora* infection.

**Table S3** Size of *pPO16::MdAGG10* lines and non-transgenic ‘Gala’ control eight weeks after greenhouse acclimatation.

**Table S4** Fire blight lesion length on *pPPO16::MdAGG10* lines 19 days after *Erwinia amylovora* infection.

**Table S5** Disease score assigned for AUDPC calculation on *in vitro* culture shoots inoculated by *Erwinia amylovora*.

**Fig. S1** T-DNA constructs and positions of gRNAs targets, quantitative PCR primers, and western-blot probes, on *MdAGG* sequences. (a) The *Cas9* gene from *Streptococcus pyogenes* is driven by *PcUbi4-2* promoter (P) from parsley (*Petroselinum crispum*) and transcription is terminated by the *Pea3a* terminator (T) from pea (*Pisum sativum*). gRNA1 and 2 are respectively driven by *MdU3* and *MdU6* promoters from *Malus domestica* and transcription is terminated by a polyT terminator (adapted from Charrier et al, 2019). (b) The *MdAGG10* gene expression from *Malus domestica* is driven by the *PPO16* promotor and transcription is terminated by the *CaMV35S* terminator. (a) & (b) Transformants are selected with a *nptII* gene controlled by *nos* promoter and terminator from *Agrobacterium tumefaciens*. *AttB1* and *2*: sites resulting from the Gateway® LR recombination. LB and RB: T-DNA borders. (c) Schematic representation, on a *MdAGG* sequence - in blue with targets of the gRNAs in green - of the position of primer pairs used in quantitative PCR to quantify the editing rate at RNA level in CRISPR lines (in yellow, pair P in Table S1) and to quantify the expression level of *MdAGG*s in CRISPR or inducible lines (in white, pair O in Table S1). (d) Schematic representation, on a MdAGG sequence of the positions of probes used to synthetize anti-MdAGG antibodies for western blot analyses.

**
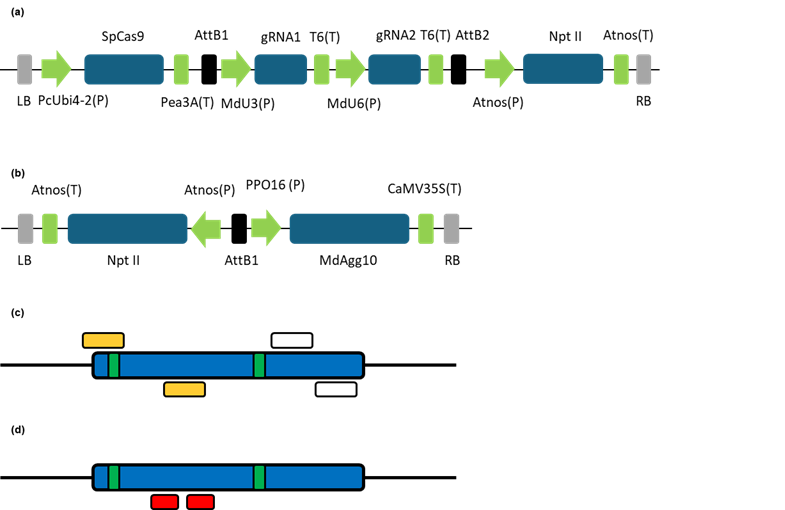
**

**Fig. S2** Genotyping of apple transgenic lines. (a) PCR on genomic DNA of the five transgenic apple lines with CRISPR/Cas9 targeting *MdAGG* sequences. Lane 1 to 5: transgenic *mdagg* lines; (b) PCR on genomic DNA of the nine transgenic apple lines with *pPPO16::MdAGG10* construct. Lane 1 to 9: pPPO16::MdAGG10 transgenic lines. AgB: Plasmid DNA extracted from the *A. tumefaciens* strain used for transformation as a positive control of T-DNA presence and a negative control of lines Agrobacterium contamination; W: water template as a PCR control ; WT : total DNA extracted from the ‘Gala’ non transformed genotype, as a negative control of T-DNA presence.

**
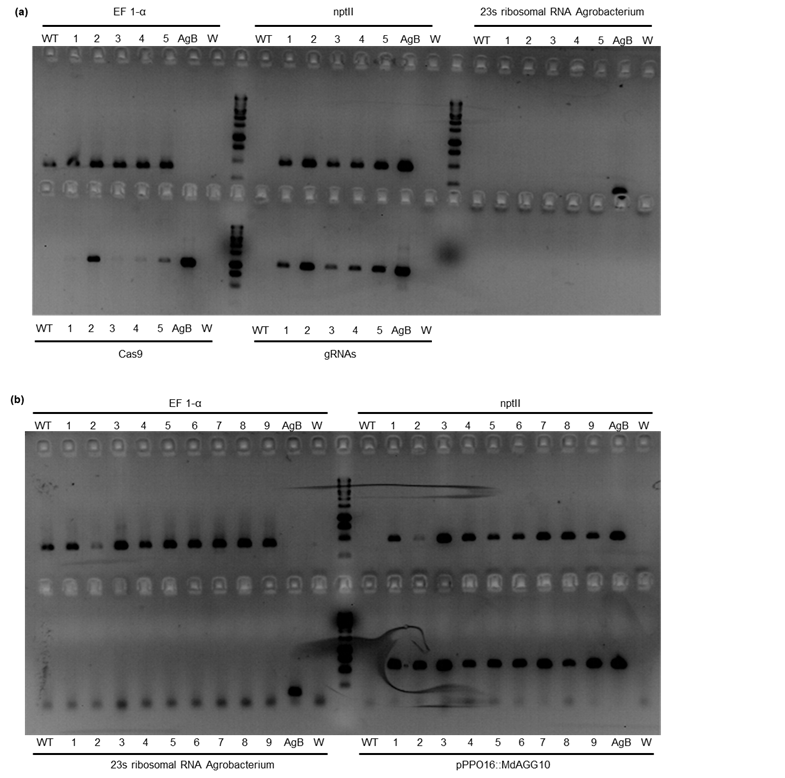
**

**Fig. S3** *MdAGGs* and *MdPPOs* expression modulation by *Ea* *T3SS* in the susceptible MM106 genotype compared with modulation by ASM in apple seedlings (Adapted from Chavonet et *al.*, 2022). Microarray analysis is represented as a scatter plot comparing the relative expressions (log 2 ratio) of apple genes in seedlings, treated three days before, with ASM relative to water treatment (x-axis, mean of four experiments), versus the relative expressions (log 2 ratio) in MM106 grafted scions, inoculated 24 h before, by *Ea* strain CFBP1430 relative to the *Ea* *t3ss* mutant (y-axis, mean of two experiments). Each scattered point represents a single apple gene. *MdAGGs* are represented by circles, *MdPPOs* by triangles. MdAGG10 and MdPPO16 locations are specified. Gray points correspond to genes that are not significantly differentially expressed in at least one of the two comparisons (P > 0.05 and –0.55< log2 ratio < 0.5).


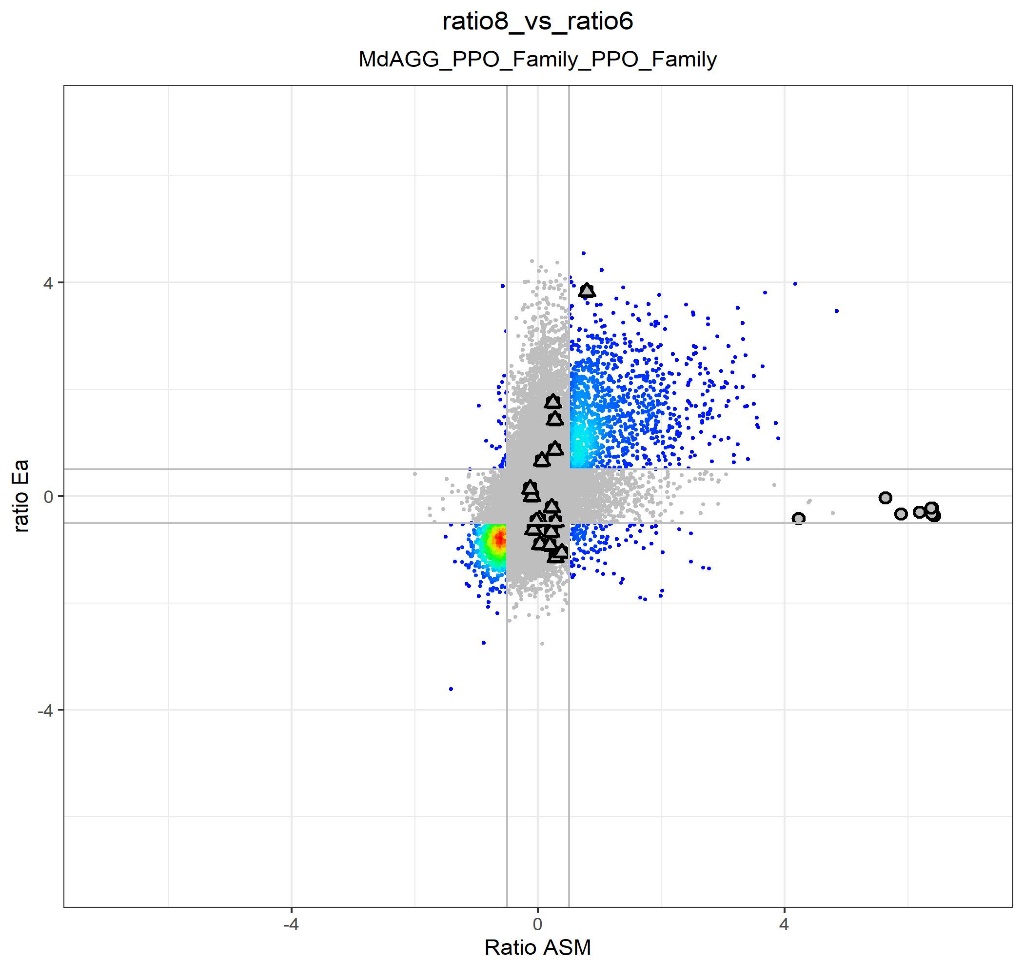


Ea strain CFBP1430 / Ea t3ss ratio

ASM / water ratio

MdAGGs

MdPPOs

MdPPO16

MdAGG10

**Table S1** Primer used in this work. * « MD » accessions are available at https://iris.angers.inra.fr/gddh13, within the "curated CDS" track.

**
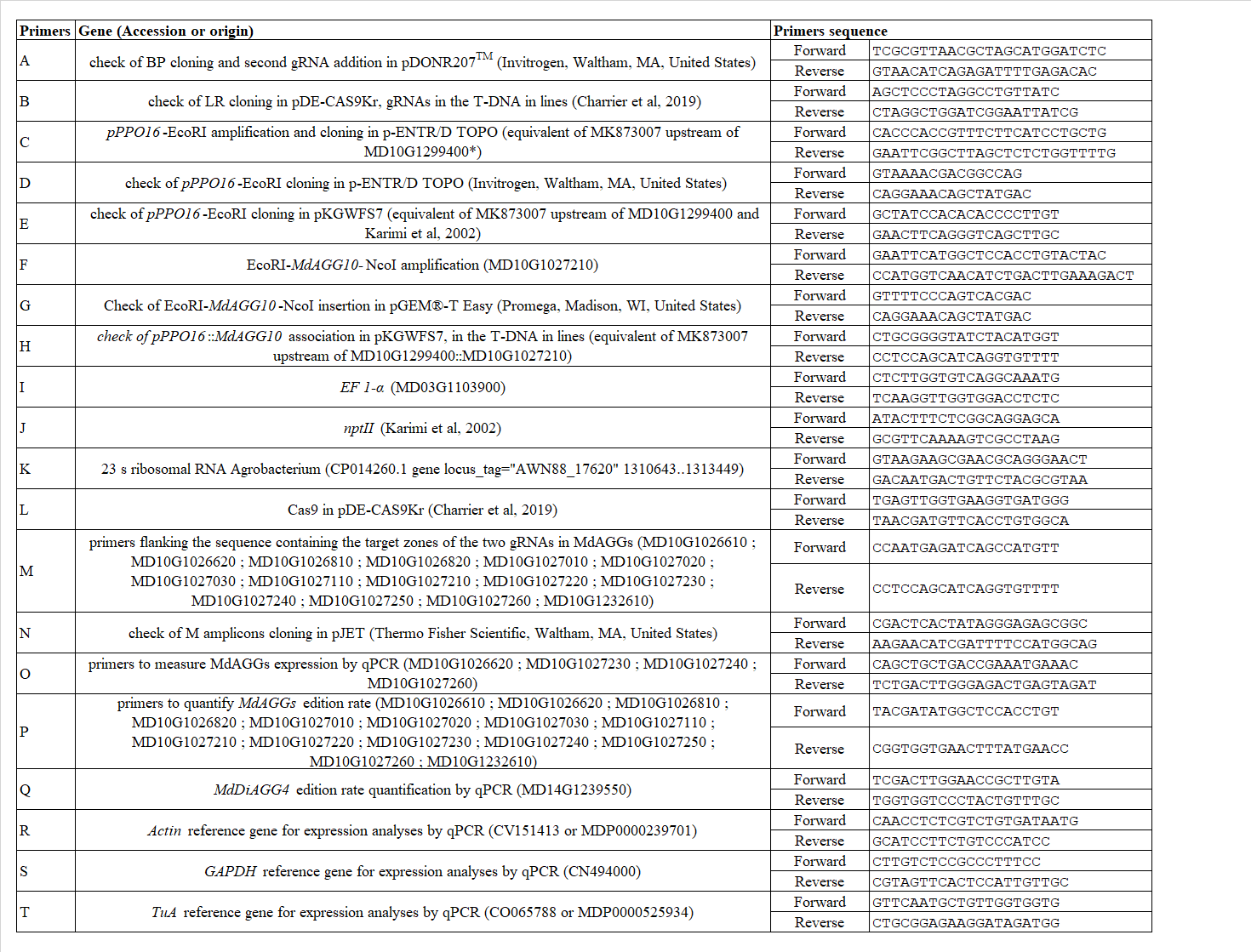
**

**Table S2** Fire blight lesion length on *mdagg* lines 21 days after *Erwinia amylovora* infection. Mean values ± standard deviation are given for all plants analysed, or separately for plants with less than 2% necrosis length or more than 2% necrosis length. Significance was assessed by plant type (all, < 2% or > 2%), using Kruskal-Wallis tests followed by Dunn's tests. Different letters indicate different statistical classes (p < 0.05).

| Genotype | ASM Treatment | Average percentage of lesion length on all plants | Percent of plant with less than 2% necrosis length | Average percentage of lesion length on plants with less thant 2% necrosis length | Average percentage of lesion length on plants with more thant 2% necrosis length |
| --- | --- | --- | --- | --- | --- |
| WT | No | 76,53 ± 39,73 (ab) | 18,18 | 0,74 ± 0,39 (a) | 93,27 ± 18,49 (a) |
| WT | Yes | 31,38 ± 43,02 (c) | 51,47 | 0,44 ± 0,52 (bc) | 78,47 ± 30,90 (a) |
| mdagg-1 | No | 94,29 ± 19,92 (a) | 2,04 | 0,86 ± 0,00 (a) | 96,24 ± 14,69 (a) |
| mdagg-1 | Yes | 66,38 ± 43,49 (b) | 27,59 | 0,72 ± 0,51 (ab) | 93,32 ± 11,02 (a) |
| mdagg-2 | No | 81,22 ± 34,66 (ab) | 16 | 0,63 ± 0,45 (ab) | 94,34 ± 11,44 (a) |
| mdagg-2 | Yes | 39,93 ± 41,71 (c) | 50 | 0,67 ± 0,38 (c) | 79,19 ± 18,49 (a) |

**Table S3** Size of *pPO16::MdAGG10* lines and non-transgenic ‘Gala’ control eight weeks after greenhouse acclimatation. Sizes were calculated on 48 non-transgenic ‘Gala’ plants, 46 *pPO16::MdAGG10-1* plants and 48 *pPPO16::MdAGG10-6* plants. Mean size ± standard deviation and significance of Tukey Test (ns: non significance).

| Plants | Size of 8 weeks-old plants (cm) |
| --- | --- |
| non transgenic 'Gala' control | 27.24 ± 2.72 (ns) |
| pPPO16::MdAGG10-1 | 28.2 ± 2.77 (ns) |
| pPPO16::MdAGG10-6 | 28.42 ± 2.24 (ns) |

**Table S4** Fire blight lesion length on *pPPO16::MdAGG10* lines 19 days after *Erwinia amylovora* infection. Mean values ± standard deviation are given for all plants analysed, or separately for plants with less than 2% necrosis length or more than 2% necrosis length. Significance was assessed by plant type (all, < 2% or > 2%), using Kruskal-Wallis tests followed by Dunn's tests. Different letters indicate different statistical classes (p < 0.05).

| Genotype | ASM Treatment | Average percentage of lesion length on all plants | Percent of plant with less than 2% necrosis length | Average percentage of lesion length on plants with less thant 2% necrosis length | Average percentage of lesion length on plants with more thant 2% necrosis length |
| --- | --- | --- | --- | --- | --- |
| WT | No | 32,90 ± 10,79 (c) | 6,06 | 1,73 ± 0,08 (c) | 34,91 ± 7,43 (a) |
| WT | Yes | 5,71 ± 6,75 (ab) | 59,26 | 0,65 ± 0,77 (b) | 13,08 ± 4,29 (ab) |
| pPPO16::MdAGG10-1 | No | 17,78 ± 15,40 (a) | 37,14 | 1,08 ± 0,54 (abc) | 27,65 ± 10,44 (a) |
| pPPO16::MdAGG10-1 | Yes | 1,81 ± 4,73 (b) | 87,88 | 0,28 ± 0,53 (ab) | 12,87 ± 7,11 (b) |
| pPPO16::MdAGG10-6 | No | 17,79 ± 17,00 (a) | 44,12 | 0,67 ± 0,66 (ac) | 31,30 ± 9,64 (ab) |
| pPPO16::MdAGG10-6 | Yes | 3,03 ± 8,96 (b) | 82,35 | 0,20 ± 0,45 (abc) | 22,79 ± 15,27 (b) |

**Table S5** Disease score assigned for AUDPC calculation on *in vitro* culture shoots inoculated by *Erwinia amylovora*.

**
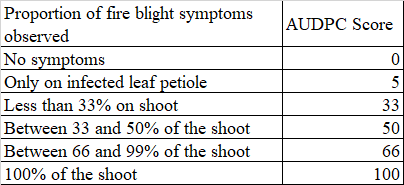
**
